# Bimodal signatures of germline methylation are linked with gene expression plasticity in the coral *Acropora millepora*

**DOI:** 10.1186/1471-2164-15-1109

**Published:** 2014-12-15

**Authors:** Groves B Dixon, Line K Bay, Mikhail V Matz

**Affiliations:** Institute for Cell and Molecular Biology, University of Texas Austin, Austin, USA; Australian Institute of Marine Science, PMB 3, Townsville, QLD 4810 Australia; ARC Centre of Excellence for Coral Reef Studies, James Cook University, Townsville, QLD 4811 Australia; Department of Integrative Biology, University of Texas Austin, Austin, USA

**Keywords:** *Acropora millepora*, Coral, DNA methylation, Gene body methylation, Gene expression, Flexibility, Plasticity

## Abstract

**Background:**

In invertebrates, genes belonging to dynamically regulated functional categories appear to be less methylated than “housekeeping” genes, suggesting that DNA methylation may modulate gene expression plasticity. To date, however, experimental evidence to support this hypothesis across different natural habitats has been lacking.

**Results:**

Gene expression profiles were generated from 30 pairs of genetically identical fragments of coral *Acropora millepora* reciprocally transplanted between distinct natural habitats for 3 months. Gene expression was analyzed in the context of normalized CpG content, a well-established signature of historical germline DNA methylation. Genes with weak methylation signatures were more likely to demonstrate differential expression based on both transplant environment and population of origin than genes with strong methylation signatures. Moreover, the magnitude of expression differences due to environment and population were greater for genes with weak methylation signatures.

**Conclusions:**

Our results support a connection between differential germline methylation and gene expression flexibility across environments and populations. Studies of phylogenetically basal invertebrates such as corals will further elucidate the fundamental functional aspects of gene body methylation in Metazoa.

**Electronic supplementary material:**

The online version of this article (doi:10.1186/1471-2164-15-1109) contains supplementary material, which is available to authorized users.

## Background

Phenotypic plasticity refers to the ability of an individual to adjust its phenotype in response to environmental cues [1]. Under changing environmental conditions, theory predicts that phenotypic plasticity may mitigate loss of fitness [2], and facilitate evolutionary adaptation [3, 4]. For sessile organisms such as plants and corals, plasticity is predicted to be of particular importance, as these organisms cannot migrate away from suboptimal environments [5, 6]. In the table top coral *Acropora hyacinthus*, colony fragments with previous exposure to elevated temperatures demonstrate increased bleaching resistance, suggesting an important role of plasticity in coral heat tolerance [7]. Hence predicting the future of reef-building corals and the ecosystems they support requires an understanding of their mechanisms of phenotypic plasticity. In most marine organisms, however, the molecular mechanisms that translate environmental stimuli into appropriate cellular responses are poorly understood [8]. One possible plasticity-modulating mechanism that is yet to be investigated in corals is DNA methylation [9–11].

DNA methylation is a widely conserved epigenetic modification involved in eukaryotic gene regulation [12]. In mammals, the majority (70-80%) of CG dinucleotides are methylated, with the exception of stretches of sequence rich in CG dinucleotides called CpG islands (CGIs) [13]. CGIs are generally not methylated, but can be targeted for methylation under particular conditions [14]. When methylation of CGIs occurs, its effect on transcription is based on the proximity of the CGI to a transcription start site (TSS), inhibiting initiation of transcription when near the TSS but not when far away from it [15]. There is evidence that this form of epigenetic regulation is involved in genome-environment interactions. DNA methylation is associated with persistent stress-induced gene expression in mice [16] and humans [17], as well as in plants [18]. It has also been linked with variation in disease development [19] and mediating phenotypic differences between monozygotic twins [20, 21].

In contrast to nearly ubiquitous methylation in mammalian genomes, genomic methylation in many invertebrates occurs specifically on CpG dinucleotides within gene bodies (also called transcription units) [22, 23]. Within gene bodies, methylation occurs primarily on exons rather than introns [24–26]. Density of gene body methylation is not equivalent across genes. Studies of multiple invertebrate taxa report bimodal patterns of gene body methylation, in which genes are separated into hypermethylated and hypomethylated classes [23, 27, 28]. Analyses of gene ontology (GO) terms in the context of these methylation classes have demonstrated characteristic divisions based on gene function. Basic biological functions with similar regulatory dynamics across tissue types and developmental stages tend toward strong methylation. Examples of basic biological functions include cellular metabolic processes, nucleic acid metabolism, and translation [28–30]. In contrast, functions that are dynamically regulated across tissues and developmental stages tend toward sparse methylation [31–33]. Examples of dynamically regulated functions include development, cell-cell signaling, and signal transduction [29, 30]. These findings suggest that bimodal gene body methylation may regulate flexibility of gene expression, with strongly methylated genes marked for stability and weakly methylated genes marked for flexibility. DNA methylation has also been linked with phenotypic plasticity, most strikingly in caste development in honeybees *Apis mellifera,* which is dependent on larval diet and activity of *de novo* DNA methyl-transferase (DNMT3) [34, 35]. Caste-specific genes in honeybees (i.e. genes with significant differential expression between queens and workers) are significantly biased toward weak methylation [31].

These findings have led to the hypothesis that invertebrate DNA methylation is involved in regulating environmentally driven gene expression and phenotypic plasticity [9, 11]. Among marine invertebrates, this hypothesis has yet to be validated in natural ecological contexts. In this study, we predicted that environmentally flexible gene expression in the branching coral *Acropora millepora* would be associated with signatures of weak gene body methylation. To test this prediction we analyzed gene expression profiles from clonal colony fragments reciprocally transplanted between distinct natural habitats. We show that elevated CpG content, a signature of historically weak germline methylation, is linked with environmentally driven gene expression. Our results suggest a potential role of DNA methylation in modulating the balance between stable gene expression required for homeostasis and flexible expression required for plasticity.

## Results

### Normalized CpG content of coding regions is bimodally distributed in *A. millepora*

Normalized CpG content (CpG_O/E_; see methods) is a well-established evolutionary signature of DNA methylation [23, 25, 28, 36]. We used this metric to estimate the strength of methylation of the coding regions of 15,221 genes across the *A. millepora* transcriptome. Because 5-methylcytosines are hypermutable [37], and invertebrate DNA methylation occurs specifically on CpG dinucleotides, sequences that are methylated in the germline are predicted to become CpG deficient over evolutionary time [38]. As a result, CpG_O/E_ values are inversely related to the degree of historical germline methylation. Low CpG_O/E_ values indicate strong methylation while high CpG_O/E_ values indicate weak methylation. Importantly, CpG_O/E_ has been shown to strongly correlate with direct measures of somatic DNA methylation in a number of animal models [23, 25, 28, 36].

**Figure 1:**
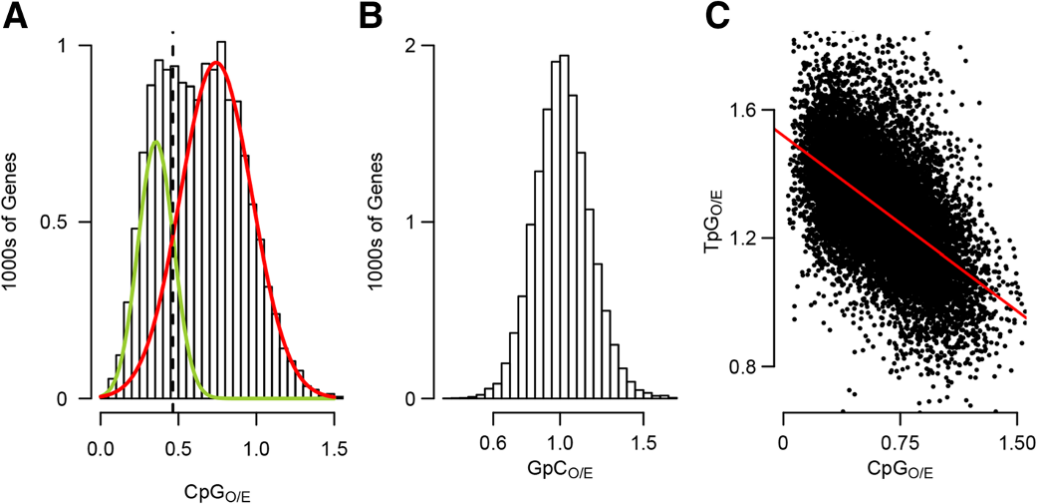
**Signatures of gene body methylation are bimodally distributed in the coral. (A)** Distribution of genes based on normalized CpG content. The green curve indicates the low-CpG component (predicted to be strongly methylated). The red curve indicates the high-CpG component (predicted to be weakly methylated). The black dotted line separates the two components at the point of intersection between the curves. **(B)** Distribution of genes based normalized GpC content. In contrast to CpG dinucleotides, GpCs are not targeted for methylation so a normal distribution is expected. **(C)** Negative linear relationship between CpG_O/E_ and TpG_O/E_. This is consistent with the prediction that DNA methylation causes depletion of CpG content largely though substitution of methylated cytosines for thymine.

Using this metric, we found a characteristic bimodal pattern in which one set of genes is predicted to have strong germline methylation and a second is predicted to have weak germline methylation. For the distribution of CpG_O/E_ values, a two-component mixture model provided substantially better fit than a single component model [Additional file 1] and as a consequence, we modeled the distribution with a two-component model (Figure 1A). We refer to the two components of the distribution as the low-CpG_O/E_ and high-CpG_O/E_ components. Means for the fitted component curves were estimated as 0.36 for the low-CpG_O/E_ component and 0.74 for the high-CpG_O/E_ component. Genes were assigned to either component based on the intersection of the fitted component curves at 0.46. Genes within the low-CpG_O/E_ component are predicted to be strongly methylated and genes in the high-CpG_O/E_ component are predicted to be weakly methylated. As a control, we showed that normalized content for GpC dinucleotides (which are not expected to be targeted for methylation) was unimodally distributed (Figure 1B). Also, as expected under the predicted mutational pattern for 5-methylcytosine (substitution for thymine as a result of deamination [37]), normalized TpG content showed an inverse linear relationship with CpG_O/E_ (Figure 1C).

### CpG_O/E_ shows characteristic associations with different biological processes

**Figure 2:**
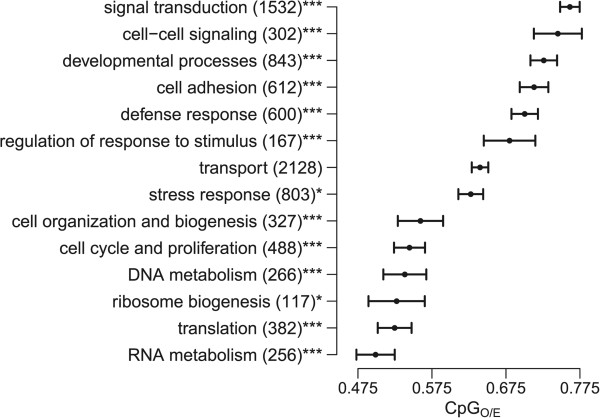
**Variation of CpG**_**O/E**_ **among genes assigned to different biological processes.** Each bar represents mean CpG_O/E_ for the indicated biological process and its standard error. Asterisks indicate significance of enrichment in the low- or high-CpG components (*< 0.05, **< 0.01, ***< 0.001; Fisher’s exact test).

To test for associations between CpG_O/E_ and gene function, we sorted genes among different biological processes based on gene ontology (GO) annotations. Mean CpG_O/E_ varied significantly between biological processes (ANOVA *p* < < 0.0001) and most processes were enriched in either the low or high-CpG component (Fisher’s exact test, Figure 2). Genes associated with RNA metabolism, translation, ribosome biogenesis, DNA metabolism, cell cycle and proliferation, and cellular organization and biogenesis tended toward low CpG_O/E_ values, indicating strong germline methylation. Genes associated with signal transduction, cell-cell signaling, developmental processes, cell adhesion, defense response and regulation of response to stimulus tended toward high CpG_O/E_, indicating weak germline methylation. Genes associated with stress response showed intermediate mean CpG_O/E_ but were enriched in the high-CpG component (*p* < 0.05).

### High CpG_O/E_ is linked with environmentally flexible gene expression

To investigate the relationship between environmentally induced gene expression and CpG_O/E_, we used DEseq [39] to compare gene expression between groups of clonal colony fragments reciprocally transplanted between two environmentally distinct natural habitats. Fifteen colonies were collected from each site and divided into two halves. One half of each colony was replaced in its native habitat, while the second half was transplanted to the alternate site (transplant location). In this way each genotype, represented by the two halves of the original colony, was exposed to both sites for a three-month study period. Following three months, global gene expression profiles were generated from all colony halves using tag-based RNA-seq method [40]. To detect effects of environment we compared gene expression between samples halves grouped by the transplantation site (the site they were placed at during the study period). In this way, the two groups being compared represented the same 30 genotypes exposed to two different environments for the three-month period. This allowed us to attribute systematic differences in gene expression between the two groups to environmental influences. To test the effect of population of origin we grouped the samples halves based on the site they originated from regardless of their transplant location during the study period. This allowed us to test for differences in gene expression that were distinctive of the two populations irrespective of environment. Expression data for each sample and estimates of differential expression between sample groups were uploaded through Zenodo at the following address [DOI] (http://dx.doi.org/10.5281/zenodo.12626) and will be available after June 1st 2015 or earlier. The read files have been uploaded the NCBI SRA (accession SRP049522) and will be released by November 11th 2015 or earlier.

**Figure 3:**
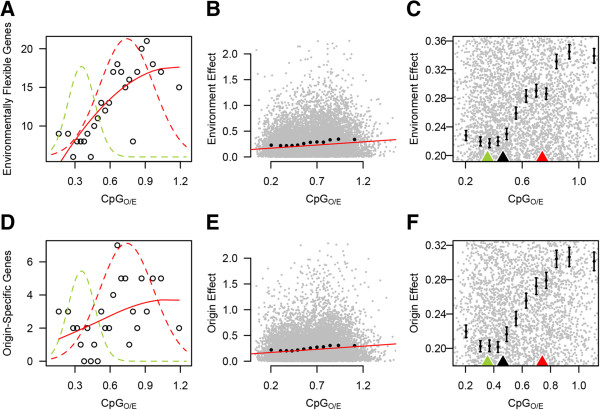
**Genes with high CpG**_**O/E**_ **are more likely to be differentially expressed between environments. (A)** Frequency of environmentally flexible genes increases with CpG_O/E_. All genes with expression data were divided into 25 quantiles based on CpG_O/E_ (503 genes per quantile). Each data point represents the count of environmentally flexible genes (adjusted *P*-value < 0.01) within a single quantile and the mean CpG_O/E_ for the quantile. To illustrate associations with the CpG_O/E_ components, the density component curves from figure 1A were traced over the count data. **(B)** Across all genes the magnitude of differential expression due to environment (environment effect) showed a positive relationship with CpG_O/E_. The red line indicates the linear model of the relationship between environmental effect and CpG_O/E_. Black error bars represent the mean and standard error for environmental effect of 12 quantiles based on CpG_O/E_. **(C)** Same as **(B)**, rescaled to illustrate that mean environment effect increases sharply under the high-CpG component. Green and red arrows along the x-axis illustrate the means for each component curve. The black arrow indicates their point of intersection. **(D-F)** Same as **A-C**, but for the effect of coral origin rather than of transplant site.

When sample halves were grouped based on transplantation site, 321 genes showed significant differential expression between the two groups (adjusted *p* < 0.01). We refer to this set of differentially expressed genes as ‘environmentally flexible genes’, since they are regulated depending on which site the sample halves were placed at. Environmentally flexible genes were significantly over-represented within the high-CpG component (Figure 3A and Table 1). Thus genes showing signatures of weak germline methylation were more likely to display environmentally driven variation in expression. To assess this relationship on a continuous scale we plotted CpG_O/E_ against the magnitude of differential expression for each gene, calculated as [log(mean expression in environment A/mean expression in environment B)]. Differential expression was positively correlated with CpG_O/E_ (Spearman's rank correlation; rho = 0.138; *p* < < 0.0001; Figure 3B). To examine if this was a simple linear relationship genes were divided into twelve quantiles based on CpG_O/E_ and mean differential expression was plotted for each quantile. This analysis revealed that the magnitude of differential expression increased sharply within the high-CpG_O/E_ component (Figure 3C), suggesting that differential expression correlates categorically with CpG_O/E_ component rather than continuously with increasing CpG_O/E_.

**Table 1:** **Enrichment of differentially expressed genes in the high-CpG component**

Effect	Effect	Low-CpG		High-CpG		***P***
	Cut-off	Significant	Not significant	Significant	Not significant	
Environment	0.1	305	3994	667	7636	3.25E-02
Environment	0.05	185	4114	494	7809	4.77E-05
Environment	0.01	67	4232	254	8049	9.72E-08
Environment	0.001	25	4274	108	8195	6.65E-05
Origin	0.1	95	4204	264	8039	9.64E-04
Origin	0.05	46	4253	149	8154	8.74E-04
Origin	0.01	13	4286	55	8248	4.87E-03
Origin	0.001	0	4299	18	8285	5.44E-04

Effect Cut-off designates the α level used to designate significant differential expression due to transplantation site (environment). *P* column designates *P-*values for enrichment of environmentally flexible genes in the high-CpG were calculated using Fisher’s exact test.

### Link between CpG_O/E_ and population-specific gene expression

As differential expression due to transplantation site showed strong bias towards high CpG_O/E_, we performed the same analyses on differential expression with respect to sample origin. Here we compared gene expression between sample halves grouped based on their site of origin. We found 68 genes that maintained origin-specific expression patterns (adjusted *p* < 0.01) irrespective of transplantation site. Differential expression due to sample origin showed similar positive associations with CpG_O/E_ (Figure 3 D-F; Table 1).

### CpG_O/E_ and gene expression level

**Figure 4:**
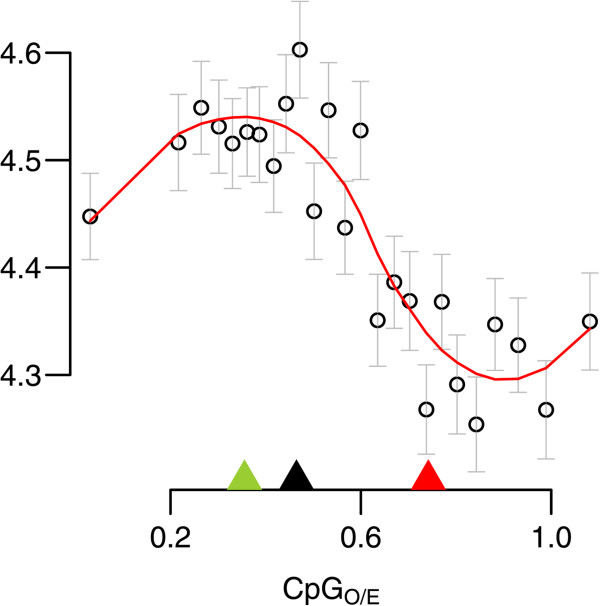
**Correlation of CpG**_**O/E**_ **with transcript abundance.** Mean gene expression values were generated from 25 equally sized quantiles based on CpG_O/E_. Each gene was assigned an expression value equal to its average expression across all samples. Each data point represents mean of the expression values for all genes included in the quantile plotted against mean CpG_O/E_ for the quantile; the whiskers denote standard errors. Green and red arrows indicate the means for the two mixture component shown in Figure 1A. The black arrow indicates the point of separation between the components.

To investigate broad scale relationships between the magnitude of gene expression and CpG_O/E_, we plotted mean transcript abundance (across all samples) for each gene against its CpG_O/E_ value (Figure 4). Genes were divided into 25 equally sized quantiles based on CpG_O/E_. Mean transcript abundance varied significantly across CpG_O/E_ quantiles (ANOVA *p* < < 0.0001) and genes in the high-CpG_O/E_ component showed decreased mean expression compared genes in the low-CpG_O/E_ component (Welch Two Sample t-test; *p* < < 0.0001).

## Discussion

### Bimodal patterns of gene body methylation as an ancestral feature among Metazoa

Depletion of CpG dinucleotides, a signature for historic germline DNA methylation, is widespread in *A. millepora* and follows a characteristic bimodal pattern. Notably, even for the high CpG_O/E_ component, the mean CpG_O/E_ value of 0.74 was less than 1.0, suggesting that these genes also bear signatures of germline methylation, although apparently weaker than genes in the low-CpG_O/E_ component. As shown by Sarda et al. [28], bimodal methylation is consistent among diverse invertebrate taxa: reported in Hymenoptera [30, 31], Hemiptera [33], Lepidoptera [36], Orthoptera [27], Mollusca [29], and Cnidaria [23, 28], This study]. Evidence of bimodal methylation in Cnidaria (the sister group to all bilaterians) along with other diverse taxa suggests an ancient mechanism that has been conserved through more than 500 million of years of evolution [41].

### Correlation between CpG_O/E_and Gene Function in *A. millepora*

Consistent with previous findings among diverse invertebrates [28–31], we found significant variation in CpG_O/E_ between different biological processes. The distribution follows a characteristic trend in which functions with spatial and temporal stability are enriched in the low-CpG_O/E_ component, implying strong methylation. Functions with greater spatial and temporal variability are enriched in the high-CpG_O/E_ component, implying weak methylation. Thus our results add to previous findings and suggest an association between weak gene body methylation and dynamic biological processes. In light of these results it was surprising that the GO term *stress response* showed an intermediate mean CpG_O/E_ and only slight enrichment in the high-CpG_O/E_ component (Figure 2). This contrasts with results from Gavery and Roberts [29], where stress response genes showed elevated CpG_O/E_. It is possible that the intermediate value for *stress response* in our system is related to regularity with which reef-building corals must contend with cellular stress. Unlike to other invertebrates examined for CpG_O/E_ previously, scleractinian corals are host to photosynthetic endosymbionts (*Symbiodinium* spp.) [42]. While the coral host depends on endosymbionts for fixed carbon, their daily photosynthetic activity causes a hyperoxic cellular environment, so that host cells are regularly exposed to oxidative stress [43]. The relatively low mean CpG_O/E_ for stress response in *A. millepora* could possibly reflect historical methylation of cellular stress response genes that require highly regular expression patterns to mediate chronic stresses of symbiosis. To support this, we compared mean CpG_O/E_ of GO terms nested within stress response and found that *oxidative stress*, *response to wounding*, and *cellular response to stress* had the lowest mean CpG_O/E_ values [see Additional file 2]. A recent comparison of gene expression in sea anemone (*Aiptasia*) revealed that along with *response to oxidative* stress, genes involved in *Inflammation, tissue remodeling,* and *response to wounding*, showed strong differential expression between symbiotic and asymbiotic individuals [44]. After removal of genes associated with *response to oxidative stress* and *cellular response to stress*, the remaining 426 stress response genes were substantially enriched in the high-CpG_O/E_ component (Fisher’s exact test *p* < 0.001). These results suggest the interesting possibility that invertebrates possess taxon-specific methylation patterns based on the demands of their particular life histories.

### Link between CpG_O/E_ and gene expression plasticity

In insects, weakly methylated status is linked with variation in gene expression across tissue types [32], caste [31], and developmental stages [25], and a similar trend across tissues types been shown in oysters (*Crassostrea gigas*) [45]. However, whether the same association is found regarding variation in gene expression across different habitats remained unresolved. Here we show that in *A. millepora*, genes with high CpG_O/E_ values are more likely to be differentially expressed in response to environmental conditions (Figure 3A and Table 1). High-CpG_O/E_ genes also show greater magnitude of expression change in response to the environment (Figure 3B-C). To the extent that CpG_O/E_ scores correlate with gene body methylation in somatic cells at the time of sampling, these results suggest a link between weak gene body methylation and gene expression plasticity.

Roberts and Gavery [11] proposed a role for gene body methylation in modulating plasticity in which weak gene body methylation passively facilitates flexible gene expression via alternative transcription start sites (TSSs), increased sequence mutations, exon skipping, or through transient gene methylation. Our results are consistent with the general hypothesis that weak gene body methylation facilitates environmentally responsive expression. To offer a potential mechanism, weak methylation could increase expression plasticity by allowing greater access to alternative TSSs. Among developmental genes in *Drosophila melanogaster*, alternative promoter use is quite common and can mediate temporally regulated expression [46]. In mammals, alternative promoter use is associated with tissue specific expression and can be regulated though methylation of intragenic CGIs [47]. Alternative TSSs could respond differently to regulatory elements and environmental cues, thereby permitting more complex and responsive transcriptional regulation. As proposed by Elango et al. [31], high CpG_O/E_ genes also could be more amenable to epigenetic modulation. Here genes that are generally weakly methylated could be conditionally methylated to mediate environmentally responsive expression. This mechanism would be of particular interest if methylation patterns were shown to fluctuate in response to environmental cues. Because CpG_O/E_ is an evolutionary signature reflecting historical methylation rather than the particular state at the time of sampling, this is a possibility that our approach could not address. An alternative hypothesis is that gene body methylation has little or no regulatory effects and is simply a byproduct of transcriptional patterns mediated by other mechanisms. Further experimentation will be required to resolve the potential explanations.

### Link between CpG_O/E_ and population-specific expression

We also identified genes with differential expression based on the corals’ origins. These genes showed expression patterns that were distinctive of the two populations and robust to changes in environmental conditions (i.e. expression differences between populations were maintained irrespective of transplant site). Our initial expectation was to find enrichment of low-CpG_O/E_ genes among these stably expressed origin-specific genes; however, we found that these genes tended toward high CpG_O/E_ similarly to the environmentally flexible genes (Figure 3D-E).

Since previous studies have demonstrated genetic structure between the Orpheus and Keppel populations [48], the simplest explanation of the origin-specific expression differences is that they result from genetic divergence. If this is the case, an intriguing possibility is that gene body methylation may help to stabilize expression patterns across divergent genetic backgrounds. Such buffering against genetic variation would be similar to the function of the heat shock protein 90 [49], except at the level of transcription rather than at the level of protein structure.

### CpG_O/E_ shows negative relationship with mean expression level

Although the difference was subtle, genes in the low-CpG_O/E_ component (indicating strong germline methylation) showed higher average expression (Figure 4). This could be attributed to the ubiquitous expression of low-CpG_O/E_ “housekeeping” genes, whereas the expression of many high-CpG_O/E_ genes is restricted to certain cell types and thus might appear lower on the scale of the whole organism. It is also possible that gene body methylation lowers gene expression noise, as has been shown in human blood and brain tissue [50], potentially leading to less frequent “off” state and thus higher average expression of low-CpG_O/E_ genes. A similar trend has been shown in the yeast (*Saccharomyces cerevisiae*) where transcriptional noise is negatively associated with protein abundance, and genes involved responding to the environment show elevated noise compared to genes involved in protein synthesis [51].

The link between predicted methylation and elevated expression contrasts with results from Riviere et al. [52], in which methylation of homeobox genes of oysters during development was inversely related to mRNA abundance. They suggest that in the case of these developmental genes a ‘CpG-island-like’ mechanism of gene repression could be responsible decreasing mRNA abundance. Hence it does not seem that gene body methylation leads to increased expression *per se* and the trend we describe is more likely due to enrichment with ubiquitously expressed housekeeping genes and not to a methylation-driven increase in expression of low-CpG_O/E_ genes.

### Corals as a model to study ecological roles of gene body methylation

Reef-building corals, as phylogenetically basal metazoans with a well-studied ecology and emerging genomic resources, represent an excellent study system to address the function of gene body methylation. Unlike most other animal models, corals can be fragmented into clonal replicates and transplanted across natural environments, making it possible to disentangle environmental and genotypic effects on genome-wide processes in realistic ecological contexts [53]. Corals can also be crossed in the lab to generate full-sib families of larvae and juveniles, which enables studies of how gene body methylation becomes established and facilitates quantitative genetic analysis [54]. Lastly, understanding the role of DNA methylation in phenotypic plasticity of corals has important conservation implications. Phenotypic plasticity is predicted to significantly influence evolutionary responses to climate change [55] and corals, the foundation of the most biologically diverse ecosystem in the ocean, are among the species most vulnerable to extinction [56].

## Conclusions

Our results indicate a connection between historical germline methylation and gene expression flexibility across environments and populations. As a whole our results are consistent with a hypothesis that strong gene body methylation leads to more stable gene expression while weak methylation facilitates flexible expression, although the direction of causality remains to be confirmed. Studies of phylogenetically basal invertebrates such as corals will further elucidate the fundamental functional aspects of gene body methylation in Metazoa.

## Methods

### Genomic resources

Coding sequences were extracted from the *A. millepora* transcriptome [57] using the script CDS_extractor.pl which is part of the Matz lab’s transcriptome annotation bundle available on the Matz lab website at http://www.bio.utexas.edu/research/matz_lab/matzlab/Methods_files/transcriptomeAssemblyAnnotation.v.1.1.tgz and on GitHub (doi:10.5281/zenodo.12232). This script uses Blastx [58] results against a protein sequences database to identify open reading frames and extract them while correcting for occasional frame shifts. For the protein reference database we combined the proteomes of *Nematostella vectensis* [59] and *Acropora digitifera* [60]. Blastx against this database was performed with an evalue cutoff of 1e-4. Based on manual verifications of a subset of *A. digitifera* protein annotations, they were pre-filtered to include sequences longer than 60 amino acids with the annotation assigned based on the listed e-value = 1e-20 or better. Annotation of our coding sequences with GO terms was based on Blastx hits to the annotated *Nematostella vectensis* [59] and *Acropora digitifera* [60] proteomes as part of the annotation pipeline indicated above. All GO annotations associated with the best hit from these references were transferred to the query sequence in our dataset. GO annotations were further supplemented with GO terms based on BLASTx matches (e-value cutoff of 10^-10^) to the SwissProt Protein database [61]. Coding sequences with no annotations were excluded from the analysis. As gene length can influence the validity of CpG_O/E_ as a predictor of methylation status [25], and DNA methylation tends to decrease toward the 3’ end of invertebrate gene bodies [23, 26], we controlled for differences in transcript length by examining CpG content only in the first 1 kb of the coding regions of each gene. Sequences shorter than 300 bp (2500 in total) were also excluded from the analysis. CpG values were bounded between 0.001 and 2, so that 1 gene with a value of 2.08 was excluded and 89 with values below 0.001 were excluded.

### CpG_O/E_ class assignment

To predict gene body methylation in *A. millepora,* we used normalized CpG content (CpG_O/E_) calculated as in Elango *et al*. [31].


Where *P*_*CpG*_*, P*_*C*_ and *P*_*G*_ are the frequencies of CpG dinucleotides, C nucleotides, and G nucleotides respectively. Because 5-methylcytosines are hypermutable [37], and invertebrate DNA methylation is targeted specifically to CpG dinucleotides, sequences that are methylated in the germline are predicted to become CpG deficient over evolutionary time [38]. As a result CpG_O/E_ is inversely related to the degree of historical germline methylation. Low CpG_O/E_ values indicate strong methylation while high CpG_O/E_ values indicate weak methylation. This metric has been shown to correlate with direct measures of DNA methylation in a number of animal models [23, 25, 28, 36].

To test whether the distribution of CpG_O/E_ values was best described as mixture of distributions we used the package Mclust [62] implemented in the R environment [63]. Bayesian information criterion (BIC) was used to compare the likelihood of Gaussian Mixture Models with different numbers of components. We found that a two-component model provided substantially better fit than a single component model [see Additional file 1 for trace of BIC for different numbers of components]. The R package Mixtools [64] was used to fit and trace the two-component mixture model of the distribution. Following Hunt et al. [33], we used the point of intersection of the two component curves to separate genes into two components: the 'low-CpG component’ (predicted to be hypermethylated) and the ‘high-CpG component’ (predicted to be hypomethylated).

### Analysis of CpG_O/E_ for biological processes

To analyze patterns of gene function relative to predicted methylation, we assigned annotated genes to 14 general biological processes based on gene ontology (GO) terms. The GO terms were selected to match a subset of those analyzed by Gavery and Roberts [29]. To these we added four additional hand-picked GO terms: *ribosome biogenesis*, *translation*, *defense response*, and *regulation of response to stimulus*, to better demonstrate the spread of “house-keeping” versus dynamic biological processes. A single gene could be assigned to multiple GO terms. GO terms with fewer than 20 representative genes, including *death* (no annotated genes) and *protein metabolism* (11 genes), were excluded from the analysis. Nonrandom sorting of genes from each function between the high-CpG_O/E_ and low-CpG_O/E_ components was ascertained using Fisher’s exact test [65].

### Reciprocal transplantation experiment

Field work was conducted with permission from the Great Barrier Reef marine Park Authority (Research permit G09/29894.1) To test for a relationship between environmentally induced gene expression and predicted methylation, we analyzed gene expression patterns of samples from a reciprocal transplantation experiment. Briefly, reciprocal transplantations were made between two environmentally distinct study sites (Keppel: 23°09S 150°54E and Orpheus 18°37S 146°29E) separated by 4.5 degrees of latitude in the Great Barrier Reef. On the 23^rd^ April (Orpheus) and 4^th^ May (Keppels) 2010 fifteen colonies were collected from wild populations from each site and divided into two. One half of each colony was replaced in its native habitat, while the second half was transplanted to the alternate study site. Samples from all coral fragments were collected at midday after three months (9^th^ July 2010 at Orpheus, 14^th^ July at Keppels) frozen in liquid nitrogen, then transferred into RNAlater (Ambion, Austin, TX, USA) for gene expression profiling.

### Analysis of gene expression

The gene expression in 56 samples (four samples failed to produce gene expression profiles and were not included) from the reciprocal transplant experiment was profiled using tag-based RNA-seq library method [40]. The latest version of the protocol and bioinformatics pipeline are available at http://sourceforge.net/projects/tag-based-rnaseq/. Briefly, the method works by sequencing short fragments from the 3’ end of mature mRNA transcripts by priming off the poly-A tail during first stand cDNA synthesis. Transcript abundance is inferred through normalized fold coverage of reads mapped to the species transcriptome [57]. While the method is exceptionally cost effective it does not allow analysis of relative expression of different transcript isoforms. Sequencing was performed using the SOLiD system producing a total of 108499924 filter passing reads (average reads per sample = 1937499 ± 788474 standard deviation). The counts data were analyzed using generalized linear model implemented in DESeq package [39] with the factors “origin” and “transplant location”. The R script used to implement DESeq is available at [DOI] (https://zenodo.org/badge/doi/10.5281/zenodo.12626.png) (http://dx.doi.org/10.5281/zenodo.12626) and on GitHub at https://github.com/grovesdixon/CpGoe.git. False discovery rate was controlled at 1% [66]. Enrichment of differentially expressed genes (i.e., showing significant effect of transplant location or origin) in the high-CpG_O/E_ component was tested using Fisher’s exact test [65]. To assess this association on a continuous scale, we plotted transplantation effect and origin effect estimated for each gene in DEseq against its CpG_O/E_. Effect size for transplantation was estimated as [log(mean expression of samples placed at Orpheus/mean expression samples placed at Keppel)], whereas the origin effect was estimated as [log(mean expression of samples that originated at Orpheus/mean expression samples that originated at Keppel)].

### Plotting relationships between expression and CpG_O/E_

To visualize genome wide trends between differential gene expression and predicted methylation (Figure 3A,D) we plotted data from 25 equal-sized gene quantiles based on CpG_O/E_. The component curves from Figure 1A were overlaid to show the relative densities of the high- and low-CpG components. Vertical scaling of the component curves did not correspond to y-axis labels. To plot mean transcript abundance against CpG_O/E_ (Figure 4), genes were divided into 25 equally sized quantiles based on CpG_O/E_ values. For each gene, expression was averaged across all samples. Mean expression for all genes in each quantile was then plotted against the mean CpG_O/E_ value for the quantile.

## Electronic supplementary material

Additional file 1: **Estimated fit for Gaussian Mixture Models.** Bayesian information criterion (BIC) was used to compare the fit of Gaussian Mixture Models with different numbers of components to the distribution of CpG_O/E_ values. BIC indicated that multicomponent models were more likely than a single component model. For simplicity, and to facilitate comparisons with previous studies we chose a two-component model. (PDF 39 KB)

Additional file 2: **Genes involved in** ***response to oxidative stress*** **and** ***cellular response to stress*** **contribute to the relatively low mean CpG**_**O/E**_ **for the** ***stress response*** **Gene Ontology term.** The figure illustrates the variation in CpG_O/E_ of Gene Ontology (GO) terms nested within stress response. Each bar represents mean CpG_O/E_ for the indicated GO term and its standard error. Asterisks indicate significance of enrichment in the low- or high-CpG components (*< 0.05, **< 0.01, ***< 0.001; Fisher’s test). The bar labelled ‘stress response reduced’ represents the *stress response* GO term with genes from *response to oxidative stress* and *cellular response to stress* removed. (PDF 59 KB)

## Abbreviations

CGIs: CpG islands
TSS: Transcription Start Site
CpG_O/E_: Normalized CpG content, specifically the CpG observed to expected ratio
GO: Gene ontology.
