# Supplementary material for: Bimodal signatures of germline methylation are linked with gene expression plasticity in the coral Acropora millepora
Source: BMC Genomics. 2014 Dec 15;15(1):1109. doi: 10.1186/1471-2164-15-1109 (PMC4378018; doi:10.1186/1471-2164-15-1109)

Estimated fit for Gaussian Mixture Models:
Bayesian information criterion (BIC) was used to compare the fit of Gaussian Mixture Models with different numbers of components to the distribution of $CpG_{O/E}$ values. BIC indicated that a two-component model provided better fit than a single component model.

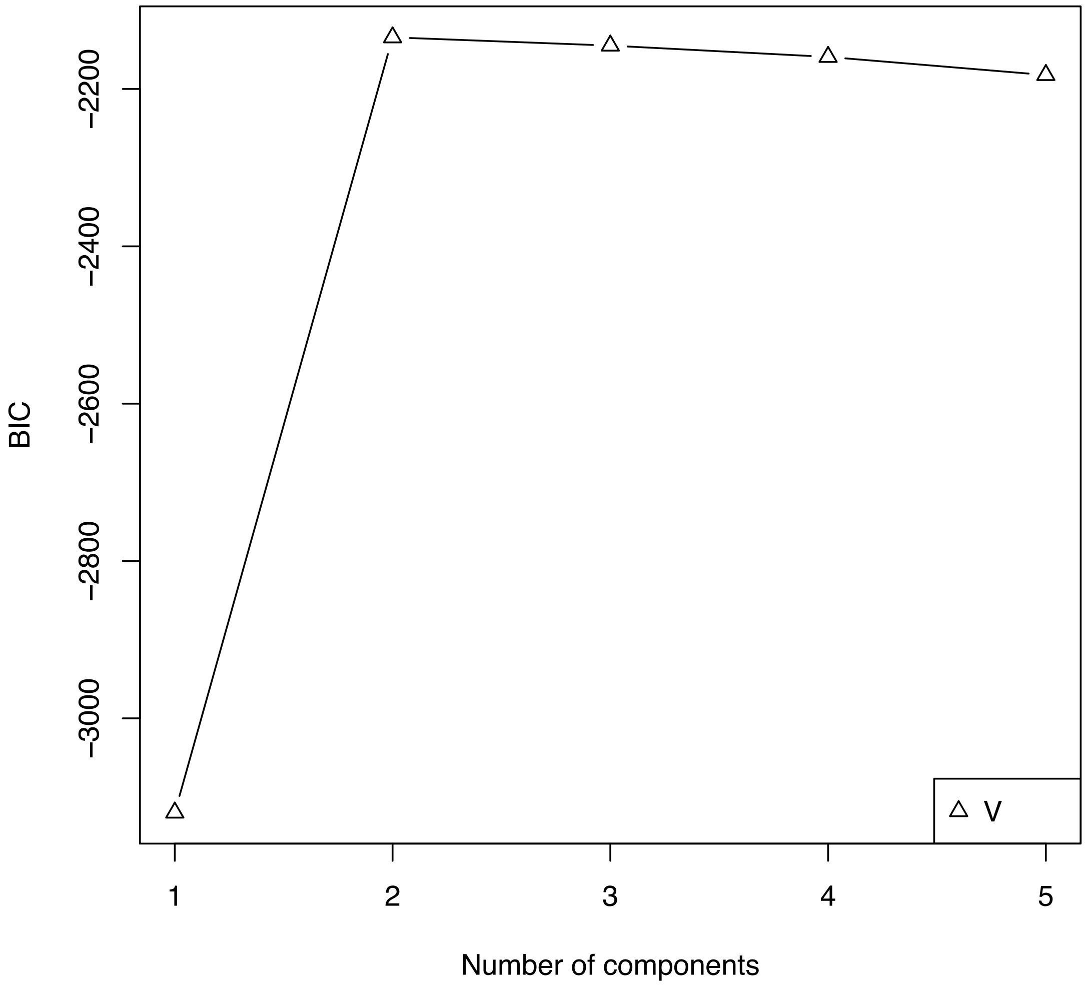

Supplement: Supplementary file 1 — Additional file 1: Estimated fit for Gaussian Mixture Models. Bayesian information criterion (BIC) was used to compare the fit of Gaussian Mixture Models with different numbers of components to the distribution of CpGO/E values. BIC indicated that multicomponent models were more likely than a single component model. For simplicity, and to facilitate comparisons with previous studies we chose a two-component model. (PDF 39 KB) [file 12864_2014_6871_MOESM1_ESM.pdf]
