# Supplementary material for: Bimodal signatures of germline methylation are linked with gene expression plasticity in the coral Acropora millepora
Source: BMC Genomics. 2014 Dec 15;15(1):1109. doi: 10.1186/1471-2164-15-1109 (PMC4378018; doi:10.1186/1471-2164-15-1109)

Genes involved in *response to oxidative stress* and *cellular response to stress* contribute to the relatively low mean $CpG_{O/E}$ for the *stress response* Gene Ontology term.

The figure illustrates the variation in $CpG_{O/E}$ of Gene Ontology (GO) terms nested within stress response. Each bar represents mean $CpG_{O/E}$ for the indicated GO term and its standard error. Asterisks indicate significance of enrichment in the low- or high-CpG components (* < 0.05, ** < 0.01, *** < 0.001; Fisher's test). The bar labeled 'stress response reduced' represents the *stress response* GO term with genes from *response to oxidative stress* and *cellular response to stress* removed. GO terms with fewer than 20 representative genes were not plotted.

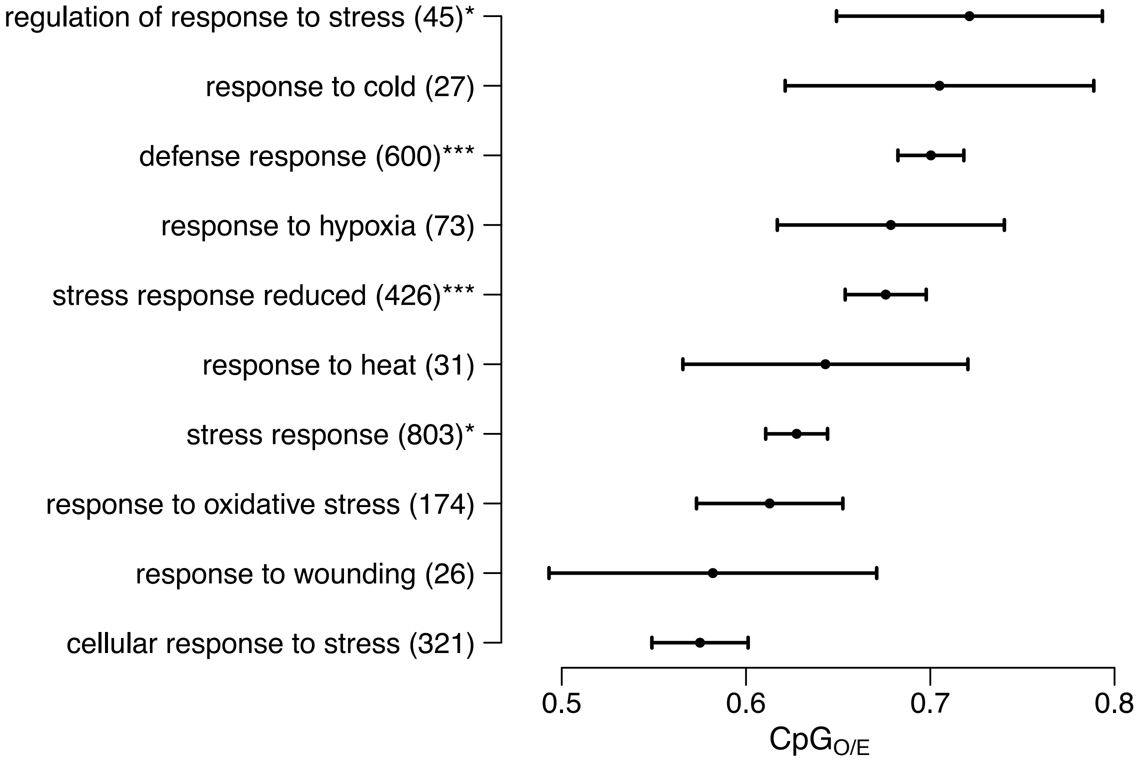

Supplement: Supplementary file 2 — Additional file 2: Genes involved in response to oxidative stress and cellular response to stress contribute to the relatively low mean CpG O/E for the stress response Gene Ontology term. The figure illustrates the variation in CpGO/E of Gene Ontology (GO) terms nested within stress response. Each bar represents mean CpGO/E for the indicated GO term and its standard error. Asterisks indicate significance of enrichment in the low- or high-CpG components (*< 0.05, **< 0.01, ***< 0.001; Fisher’s test). The bar labelled ‘stress response reduced’ represents the stress response GO term with genes from response to oxidative stress and cellular response to stress removed. (PDF 59 KB) [file 12864_2014_6871_MOESM2_ESM.pdf]
